# Aortic Valve Annular Morphology in Healthy Adults With Left Ventricular ‘Rigid Body Rotation’: A Detailed Three-Dimensional Speckle-Tracking Echocardiographic Analysis From the MAGYAR-Healthy Study

**DOI:** 10.31083/RCM48392

**Published:** 2026-07-17

**Authors:** Attila Nemes, Nóra Ambrus, Csaba Lengyel

**Affiliations:** ^1^Department of Medicine, Albert Szent-Györgyi Medical School, University of Szeged, H-6725 Szeged, Hungary

**Keywords:** aortic valve, three-dimensional, left ventricular, speckle-tracking, echocardiography

## Abstract

**Background:**

Due to the specific arrangement of left ventricular (LV) muscle fibers, the apex and base of the LV rotate in opposite directions under healthy conditions, resulting in LV twist. However, in certain, primarily pathological, conditions, LV twist is absent, and the base and apex of the LV rotate in the same direction, either counterclockwise (CCW) or clockwise (CW); this motion is known as LV rigid body rotation (RBR). In the literature, no clear associations have currently been identified between LV rotational parameters and aortic valve annulus (AVA) dimensions in healthy adults. Thus, this study aimed to compare AVA dimensions in the presence of LV-RBR with those in cases with normally directed LV rotational mechanics (ND-LVrot) under healthy conditions.

**Methods:**

This investigation included 110 healthy subjects, of whom 100 had ND-LVrot (mean age: 35.6 ± 12.0 years; 61 males) and 10 had LV-RBR (mean age: 35.4 ± 11.3 years; 7 males). The presented investigation is part of the “Motion Analysis of the heart and Great vessels bY three-dimensionAl (3D) speckle-tRacking echocardiography (3DSTE) in Healthy subjects” (MAGYAR-Healthy) Study.

**Results:**

No differences in AVA dimensions were present between individuals with ND-LVrot and those with LV-RBR at the group level. However, CCW-LV-RBR cases exhibited dilated end-systolic AVA dimensions compared with both ND-LVrot and CW-LV-RBR subjects. AVA plane systolic excursion (AAPSE) was significantly impaired in CW-LV-RBR cases compared with CCW-LV-RBR cases. Basal LV strains did not differ significantly among healthy cases with ND-LVrot, CW-LV-RBR, and CCW-LV-RBR.

**Conclusions:**

Although AVA dimensions and AAPSE did not differ between ND-LVrot and LV-RBR cases at the overall group level, significant differences in AVA dimensions and AAPSE were observed between LV-RBR cases with CW and CCW rotational directions.

## 1. Introduction

The left ventricle (LV) is the engine of the central circulation, whose pumping activity can be characterized by numerous parameters. Owing to the unique orientation of the LV myocardial fibers, the base and apex rotate in opposite directions under physiological conditions, generating the phenomenon of LV twist [1,2,3,4,5,6,7,8,9,10]. However, in certain, primarily pathological, conditions, LV twist is absent, and the LV apex and base rotate in the same direction, either counterclockwise (CCW) or clockwise (CW), a phenomenon known as LV rigid body rotation (RBR) [8,9,10,11]. Notably, according to the literature, LV-RBR is also observed in approximately 6% of healthy individuals [12].

The aortic valve serves as the anatomical boundary between the LV and the aorta and consists of a fibrous annulus (aortic valve annulus (AVA)) and three thin semilunar leaflets [13,14]. In a recent study, no clear associations were found between LV rotational features and AVA size in healthy individuals [15]. However, one question remained unanswered: whether differences in AVA sizes and AVA plane systolic excursion (AAPSE), which reflects the associated longitudinal excursion within the three-dimensional (3D) space, are observed in the presence of LV-RBR. The primary advantage of 3D speckle-tracking echocardiography (3DSTE) is the associated capacity of the approach to quantify LV rotational mechanics simultaneously, and AVA dimensions, for which established normal reference values are available [12,16,17,18,19]. Therefore, this study aimed to evaluate how LV-RBR affects AVA dimensions and AAPSE by comparing healthy individuals exhibiting this mechanics with healthy controls with normally directed LV rotational mechanics (ND-LVrot).

## 2. Subjects and Methods

### 2.1 Population of Healthy Subjects

This investigation included 110 healthy subjects, of whom 100 had ND-LVrot (mean age: 35.6 ± 12.0 years; 61 males), while 10 had LV-RBR (mean age: 35.4 ± 11.3 years; 7 males). Participants were voluntarily enrolled between 2011 and 2017. Each subject underwent a comprehensive clinical evaluation, including physical examination, laboratory screening, 12-lead electrocardiography (ECG), and standard two-dimensional (2D) Doppler echocardiography supplemented with 3DSTE. All findings were negative, with parameters falling within established normal reference ranges. Furthermore, no known disorders, pathologies, or abnormalities were reported in the medical histories of the participants. Moreover, none of the participants were regular medication users, smokers, athletes, pregnant, or obese (body mass index >30 kg/m^2^). The present investigation is part of the “Motion Analysis of the heart and Great vessels bY three-dimensionAl speckle-tRacking echocardiography in Healthy subjects” (MAGYAR-Healthy) Study (the term “Magyar” refers to “Hungarian” in the Hungarian language), which partly aimed to investigate cardiac physiology under healthy conditions. The study was approved by the Institutional and Regional Human Biomedical Research Committee of the University of Szeged, Hungary (registration number 71/2011), with the latest approval granted on 17th March, 2025. This research adhered to the 2013 revision of the Declaration of Helsinki, and all subjects provided written informed consent before enrollment.

### 2.2 Two-dimensional Doppler Echocardiography

All echocardiographic analyses were performed using a Toshiba Artida® ultrasound system (Toshiba Medical Systems, Tokyo, Japan) equipped with a PST-30BT (1–5 MHz) phased-array probe. The diagnostic protocol comprised assessment of LV dimensions and LV ejection fraction (EF) via the modified Simpson biplane method, as well as measurement of left atrial (LA) dimensions. Additionally, Doppler echocardiography was used to exclude significant valvular pathology and to measure E and A wave velocities [20].

### 2.3 Three-dimensional Speckle-tracking Echocardiography

The 3DSTE analysis was performed using a two-step process [16,17,18,19]. Initially, 3D echocardiographic datasets were digitally acquired with a Toshiba Artida® system equipped with a 3D-capable PST-25SX matrix-array transducer. To optimize image quality, imaging parameters, including magnitude and gain, were meticulously adjusted before digital acquisition of 3D datasets from the apical window. To maintain stable R-R intervals, data were obtained during held end-expiration in all healthy participants. A full volume dataset was then generated by automatically stitching together six subvolumes acquired over six consecutive heart cycles.

In the second step, offline analysis was performed using dedicated, vendor-specific software (version 2.7, 3D Wall Motion Tracking, UltraExtend; Toshiba Medical Systems, Tokyo, Japan). Simultaneous analyses included LV and AVA. Image quality was qualitatively assessed based on the clarity of the endocardial border definition across all segments in all cases. Datasets with significant acoustic dropout or stitching artifacts that impeded tracking were excluded from the analysis. Overall image quality was graded using a 5-point Likert scale (1: poor, 5: excellent), with a minimum score of 3 required for inclusion.

**Fig. 1.**
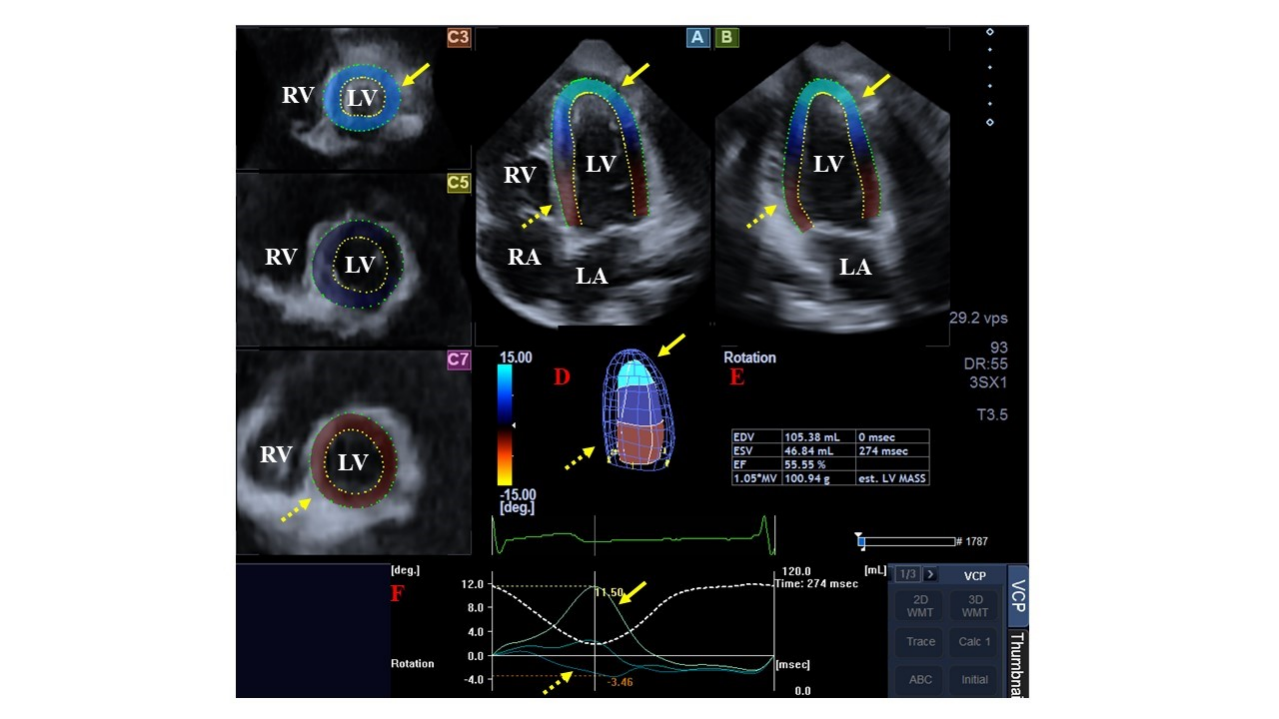
**Assessment of the left ventricular rotational mechanics by three-dimensional speckle-tracking echocardiography**. Quantification of left ventricular (LV) rotational mechanics via three-dimensional speckle-tracking echocardiography (3DSTE) in a healthy subject presents apical four-chamber (A) and two-chamber (B) long-axis views, along with short-axis views at basal (C3), midventricular (C5), and apical (C7) LV levels. These are shown alongside a 3D LV cast (D) and calculated LV volumes (E), while LV apical (yellow arrow) and basal (dashed yellow arrow) regions with corresponding time-LV rotation curves (coloured curves) are presented with the time–LV volume change curve (dashed white curve) during the heart cycle (F). **Abbreviations**: ESV, end-systolic volume; EDV, end-diastolic volume; EF, ejection fraction; RV, right ventricle; RA, right atrium; LV, left ventricle; LA, left atrium.

For LV assessment, the software generated a 3D LV model/cast, from which LV rotational parameters and volumes were measured. The software automatically generated apical 2-chamber (AP2CH) and 4-chamber (AP4CH) long-axis LV views, as well as apical, midventricular, and basal LV cross-sectional views. After manual definition of the edges of the septal and lateral LV and mitral annulus (MA) and endocardium of the LV apex, a sequential analysis was performed. LV twist was calculated as the net difference between basal and apical LV rotations (Fig. 1) [12]. In cases of LV-RBR, where physiological LV twist is absent, the LV apico-basal gradient was calculated as the difference between LV rotations in the same direction (CW or CCW) [11]. Additionally, basal segmental longitudinal (LS), circumferential (CS), and radial (RS) strains were measured to assess LV thickening/thinning, shortening/lengthening, and narrowing/widening of the LV [21,22,23].

**Fig. 2.**
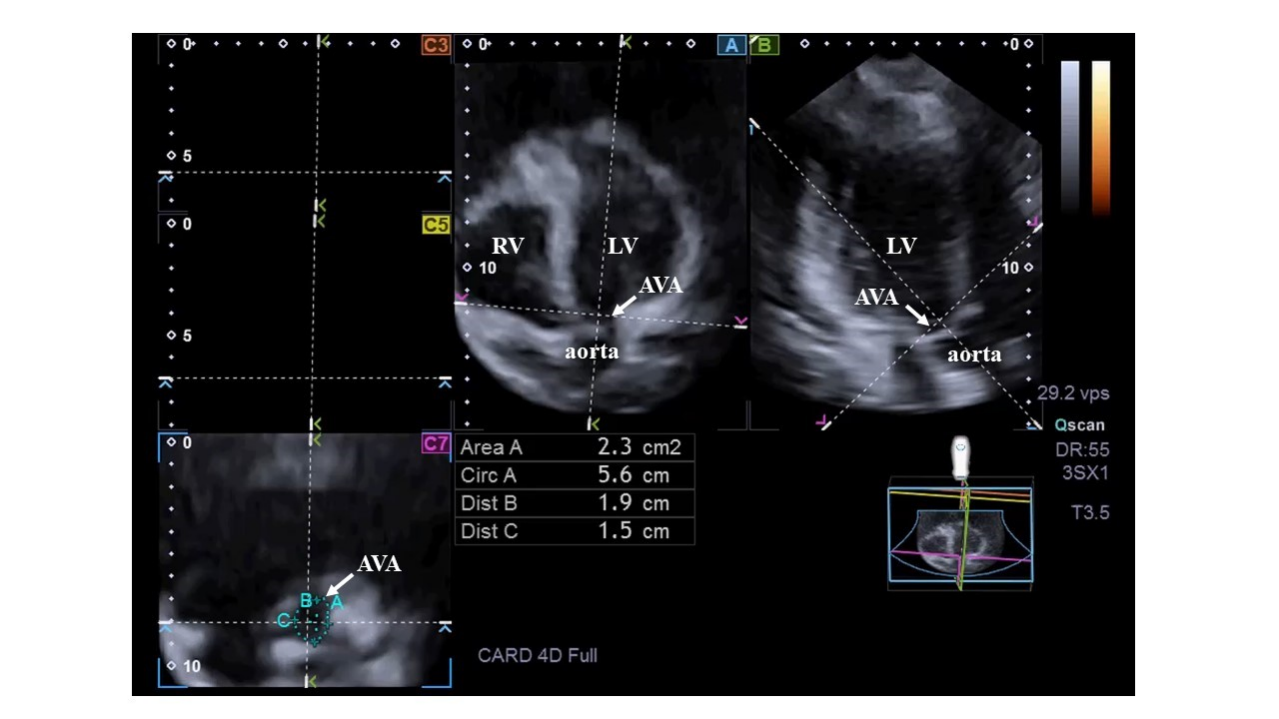
**Evaluation of aortic valve annular geometry via three-dimensional speckle-tracking echocardiography**. Assessment of aortic valve annular (AVA) dimensions in apical four-chamber (A) and two-chamber (B) long-axis views by three-dimensional speckle-tracking echocardiography. ‘En-face’ view of the AVA is also presented in C7 cross-sectional view. **Abbreviations:** LV, left ventricle; Dist B and C, maximum and minimum AVA diameters; Circ, AVA perimeter; area, AVA area; AVA, aortic valve annulus.

AVA dimensions were determined using AP4CH and AP2CH long-axis views, ensuring that the longitudinal planes were oriented parallel to the midline of both the aortic root and aortic valve. The C7 cross-sectional view, positioned perpendicular to this plane, was used to measure end-diastolic and end-systolic dimensions, including the minimum and maximum AVA diameters (AVA-Dmin and AVA-Dmax, respectively), AVA perimeter (AVA-P), and AVA area (AVA-A), all obtained from planimetric images. Precise care was taken to maintain the C7 cross-sectional view strictly perpendicular to the longitudinal axis, thereby preventing measurement errors associated with the inclusion of the sinuses of Valsalva or the LV outflow tract. Additionally, the spatial displacement of the AVA plane throughout the cardiac cycle, referred to as AAPSE, was also quantified (Fig. 2) [24,25,26].

### 2.4 Statistical Analysis

Results are reported as the mean ± standard deviation for continuous variables and as n (%) for categorical variables, as appropriate. A *p*-value < 0.05 was considered statistically significant. Normality of continuous variables, such as AVA dimensions and AAPSE, was assessed using the Shapiro–Wilk test, and homogeneity of variances was assessed using Levene’s test. Depending on the data type, Fisher’s exact test or Student’s *t*-test was employed for categorical and continuous variables, respectively. Intra-observer and inter-observer agreements were tested by intraclass correlation coefficients (ICCs). Data were analyzed using the SPSS statistical package, version 29.0.0.0 (IBM Corp., Armonk, NY, USA).

## 3. Results

### 3.1 Clinical Data and 2D Doppler Echocardiography

This study includes a healthy population that overlaps in part with cohorts described in prior publications from the MAGYAR-Healthy Study. Clinical data are presented in Table 1. All 2D echocardiographic metrics were within normal limits, including parameters such as LA diameter (37.3 ± 3.8 mm), end-diastolic and end-systolic LV diameters (48.2 ± 3.8 mm and 32.1 ± 3.2 mm, respectively) and volumes (107.1 ± 23.8 mL and 38.1 ± 9.3 mL, respectively), interventricular septal thickness (9.3 ± 1.3 mm), LV posterior wall thickness (9.5 ± 1.5 mm), and LV-EF (64.7 ± 4.0%). Diastolic transmitral E and A inflow velocities were 78.2 ± 16.7 cm/s and 59.3 ± 14.2 cm/s, respectively. None of the individuals exhibited valvular regurgitation of grade 1 or higher, or any significant valvular stenosis. Visual assessment confirmed the absence of wall motion abnormalities in all subjects.

**Table 1.** **Clinical and demographic data of healthy individuals with a normal pattern of left ventricular twist were compared with those of individuals with rigid body rotation of the left ventricle**.

	All healthy subjects	ND-LVrot	All LV-RBR	CCW-LV-RBR	CW-LV-RBR
n	110	100	10	5	5
Male gender (%)	68 (62)	61 (61)	7 (70)	5 (100)	2 (40)
Age (years)	35.6 ± 12.0	35.6 ± 12.0	35.4 ± 11.3	40.4 ± 12.6	30.4 ± 6.8
Weight (kg)	75.3 ± 17.9	75.2 ± 18.0	76.1 ± 16.8	82.7 ± 5.7	71.3 ± 20.3
Height (cm)	173.2 ± 12.2	173.0 ± 12.5	175.6 ± 8.1	182.3 ± 3.9	170.5 ± 6.5
Body surface area (m^2^)	1.92 ± 0.21	1.92 ± 0.20	1.94 ± 0.27	2.08 ± 0.07	1.86 ± 0.31
Body mass index (kg/m^2^)	24.1 ± 2.0	24.0 ± 1.7	24.8 ± 2.2	25.0 ± 2.1	24.6 ± 2.3
Blood pressure at systole (mm Hg)	121.0 ± 4.3	121.3 ± 4.0	120.9 ± 4.1	120.4 ± 4.5	121.4 ± 4.0
Blood pressure at diastole (mm Hg)	73.1 ± 3.5	73.3 ± 3.8	73.0 ± 3.3	72.8 ± 3.1	73.1 ± 3.5
Heart rate (bpm)	71.2 ± 2.5	70.9 ± 2.3	71.3 ± 2.5	71.1 ± 2.3	71.5 ± 2.6

**Abbreviations**: LV, left ventricular; ND-LVrot, normally directed left ventricular rotational mechanics; RBR, rigid body rotation; CCW, counterclockwise; CW, clockwise.

### 3.2 Quality of 3DSTE-derived Datasets

The quality distribution of the included 3D datasets was as follows: 35% excellent, 40% good, and 25% fair. The mean image quality score was 3.8 ± 0.7, indicating a robust dataset for parameter optimization.

### 3.3 Statistical Tests

Normality and homogeneity tests confirmed that the AVA dimensions and AAPSE were normally distributed (Shapiro–Wilk test), justifying the use of independent *t*-tests.

### 3.4 AVA Parameters

AVA-A was greater in end-diastole in 29 cases (29%) and in end-systole in 62 subjects (62%), while end-diastolic and end-systolic AVA-A values were equal in nine cases (9%) among healthy individuals with ND-LVrot. In 10 individuals with LV-RBR, three (30%) had a larger AVA-A in end-diastole, a proportion similar to that in subjects with ND-LVrot (29% vs. 30%; *p* = ns). However, all three of these LV-RBR individuals had CW-LV-RBR. No differences in AVA dimensions were found between subjects with ND-LVrot and LV-RBR cases at the group level. Nonetheless, CCW-LV-RBR cases showed dilated end-systolic AVA dimensions compared with both CW-LV-RBR cases and individuals with ND-LVrot. AAPSE was significantly lower in CW-LV-RBR cases than in those with CCW-LV-RBR. Basal LV strains did not differ significantly among healthy individuals with ND-LVrot, CW-LV-RBR, and CCW-LV-RBR (Table 2).

**Table 2.** **Assessment of annular sizes of the aortic valve, longitudinal displacement, and regional deformation of the left ventricle in healthy individuals with a normal pattern of left ventricular twist compared to those with rigid body rotation of the left ventricle**.

	All healthy subjects (n = 110)	ND-LVrot (n = 100)	All LV-RBR (n = 10)	CCW-LV-RBR (n = 5)	CW-LV-RBR (n = 5)
AVA-Dmax-D (cm)	2.01 ± 0.31	2.01 ± 0.29	2.05 ± 0.43	2.12 ± 0.21	1.98 ± 0.56
AVA-Dmin-D (cm)	1.81 ± 0.29	1.82 ± 0.29	1.78 ± 0.29	1.90 ± 0.14	1.66 ± 0.34
AVA-A-D (cm^2^)	3.12 ± 0.84	3.12 ± 0.82	3.10 ± 0.96	3.40 ± 0.48	2.80 ± 1.19
AVA-P-D (cm)	6.29 ± 0.86	6.29 ± 0.83	6.26 ± 1.12	6.62 ± 0.41	5.90 ± 1.44
AVA-Dmax-S (cm)	2.05 ± 0.29	2.04 ± 0.29	2.11 ± 0.25	2.28 ± 0.15	1.94 ± 0.21
AVA-Dmin-S (cm)	1.86 ± 0.28	1.86 ± 0.27	1.95 ± 0.28	2.12 ± 0.15*†	1.78 ± 0.28
AVA-A-S (cm^2^)	3.34 ± 0.85	3.32 ± 0.84	3.54 ± 0.88	4.12 ± 0.53*††	2.96 ± 0.78
AVA-P-S (cm)	6.49 ± 0.84	6.47 ± 0.83	6.71 ± 0.87	7.28 ± 0.45**†††	6.14 ± 0.81
AVA-A is larger in end-diastole	32 (29)	29 (29)	3 (30)	0 (0)†††	3 (60)
AAPSE (cm)	1.16 ± 0.30	1.15 ± 0.31	1.18 ± 0.13	1.28 ± 0.07††††	1.08 ± 0.10
Basal LV-RS (%)	32.7 ± 11.9	32.7 ± 12.1	33.3 ± 9.8	33.7 ± 9.8	33.0 ± 9.9
Basal LV-CS (%)	–26.0 ± 5.0	–26.0 ± 5.1	–26.3 ± 3.5	–25.2 ± 4.7	–27.5 ± 0.6
Basal LV-LS (%)	–20.3 ± 4.4	–20.3 ± 4.4	–20.8 ± 4.6	–22.0 ± 4.5	–19.6 ± 4.4

*p = 0.04 vs. ND-LVrot, **p = 0.03 vs. ND-LVrot, †*p* = 0.02 vs. CW-LV-RBR, ††*p* = 0.05 vs. CW-LV-RBR, †††*p* = 0.04 vs. CW-LV-RBR, ††††*p* = 0.01 vs. CW-LV-RBR.** Abbreviations**: ND-LVrot, normally directed left ventricular rotational mechanics; AVA, aortic valve annulus; AAPSE, AVA plane systolic excursion; Dmin, minimum diameter; Dmax, maximum diameter; P, perimeter; A, area; D, end-diastolic; S, end-systolic; LV, left ventricular; RS, radial strain; CS, circumferential strain; LS, longitudinal strain; RBR, rigid body rotation; CCW, counterclockwise; CW, clockwise.

### 3.5 Intra- and Inter-observer Variability of 3DSTE-derived Variables

Intra-observer ICCs for AVA-Dmax-D, AVA-Dmin-D, AVA-A-D, AVA-P-D, AVA-Dmax-S, AVA-Dmin-S, AVA-A-S, AVA-P-S, AAPSE, basal LV-RS, basal LV-CS, and basal LV-LS were 0.86, 0.90, 0.95, 0.92, 0.92, 0.81, 0.91, 0.92, 0.92, 0.83, 0.83, and 0.82, respectively. Inter-observer ICCs for the same parameters were 0.87, 0.92, 0.95, 0.93, 0.93, 0.83, 0.93, 0.93, 0.92, 0.80, 0.79, and 0.79, respectively.

## 4. Discussion

The physiological hallmark of LV rotational mechanics is that, in healthy subjects, the apex rotates CCW and the base rotates CW during the cardiac cycle [6,7,8,9,10]. This counter-directional motion, analogous to wringing out a towel, generates LV twist, which is defined as the net difference between apical and basal LV rotations. This motion accounts for approximately 40% of ejection; the associated physiological basis lies in the crossed-helical architecture of the myocardium, specifically the opposing orientation of the oblique fibers: the subendocardial fibers follow a right-handed helix, whereas the subepicardial fibers form a left-handed helix. This arrangement creates the mechanical leverage necessary for efficient LV twisting [1,2,3,4,5,6,7,8,9,10]. However, in several pathologies, LV rotational mechanics may be absent or abnormal, such that either the LV base or the LV apex moves in a direction different from the physiological direction [11]. This phenomenon is called LV-RBR and can be CW- or CCW-directed. Movement of the LV base in the CCW direction is termed CCW-LV-RBR, whereas CW rotation of the LV apex is referred to as CW-LV-RBR [11]. According to the literature, LV-RBR is most commonly observed in the presence of LV noncompaction, cardiac amyloidosis, and acromegaly, but it is also present in approximately 6% of healthy individuals [12]. Current echocardiographic guidelines clearly recommend 3DSTE for determining this motion [27], whose normal reference values have also been defined; the associated age- and gender-dependent nature has been demonstrated [12]. Overall, 3DSTE is a validated and useful procedure for determining LV rotational parameters [28,29,30,31,32].

The AVA is an essential component of the aortic valve and is closely related to both the aorta and the adjacent LV [13,14]. Approximately 60% of subjects exhibit a larger end-systolic AVA-A, whereas the end-diastolic AVA-A is greater in about 30% of cases [25,33]. The clinical utility of 3D echocardiography for determining AVA dimensions is well-established, and standardized normal reference values are available for 3DSTE-derived parameters [25,34]. A recent study found no clear association between ND-LVrot and AVA dimensions [15]. However, whether these findings are preserved in healthy individuals exhibiting LV-RBR remains unclear.

The findings of this study have several implications. First, both LV rotational mechanics, including LV-RBR, and AVA dimensions can be evaluated simultaneously from the same 3D datasets in physiological studies, as previously demonstrated in the MAGYAR-Healthy Study. Second, the proportion of individuals with a greater end-diastolic AVA-A was similar between healthy subjects with ND-LVrot and those in the LV-RBR group. However, all LV-RBR in this context proved to be CW-oriented. Third, AVA dimensions and the spatial displacement of the AVA throughout the heart cycle, as represented by AAPSE, were similar in LV-RBR and ND-LVrot cases at the group level. CCW-LV-RBR cases showed dilated end-systolic AVA dimensions compared with both CW-LV-RBR cases and subjects with ND-LVrot. AAPSE was significantly reduced in CW-LV-RBR cases compared with subjects with CCW-LV-RBR. Fourth, no differences in basal LV strain were observed between the healthy subgroups examined. From the perspective of a clinician, the most important finding is that 3DSTE allows not only detailed analysis of LV rotational mechanics, with simultaneous determination of AVA dimensions and displacement, but also analysis of the relationship between them. Meanwhile, LV-RBR can be present even in seemingly healthy individuals and may be associated with functional LV abnormalities. Moreover, although under normal conditions the base of the LV rotates CW onto the AVA during end-systole, according to this study, in the case of CCW-LV-RBR, the opposite rotation occurs. In the presence of such counter-directional basal LV rotation, the LV can maintain adequate pump function—as represented by normal functional parameters—only at the expense of AVA expansion. Thus, whether this has late-term consequences for the development of any aortic valve pathology remains uncertain. These findings suggest differences between CCW- and CW-oriented LV-RBR in AVA dimensions and function in healthy individuals. However, further studies in larger healthy populations are required to validate the present results using other advanced cardiovascular imaging techniques.

### Limitations

The following limitations were identified during the analytical process:

The most important limitation of 3DSTE is image quality, which is generally lower than that of 2D echocardiography because 3D acquisition requires 4–6 cardiac cycles, the probe is relatively large, and respiratory motion or arrhythmias can also affect image quality [16,17,18,19].

Before conducting the study, it would have been important to perform a statistical power analysis. However, only a limited number of healthy subjects with LV-RBR (n = 10) were included in the present study. While the true prevalence of LV-RBR in the general healthy population remains unknown, data from the MAGYAR-Healthy Study indicate a prevalence of approximately 6% [12]. Increasing the number of healthy cases with LV-RBR would have improved the power of the statistical analysis; however, such cases are not feasible due to the rarity of this phenomenon. Despite the inclusion of healthy subjects in this study, subclinical abnormalities cannot be entirely excluded. Moreover, although all other cardiac chambers and valvular annuli can be examined simultaneously, only the LV and AVA were investigated. Validation of 3DSTE-derived rotational parameters was not an objective of this study, given the previously established validity of these parameters [28,29,30]. Finally, in this study, the ventricular septum was assigned to the LV, although it remains debatable whether the ventricular septum belongs to the left or right ventricle.

## 5. Conclusions

Although no differences in AVA dimensions and AAPSE were observed between subjects with ND-LVrot and individuals with LV-RBR at the group level, significant differences in AVA dimensions and AAPSE were found between CW-LV-RBR and CCW-LV-RBR cases.

## Data Availability

The data sets generated and analyzed during the current study are not publicly available due to local restrictions, but are available from the corresponding authors on reasonable request.
